# Antibacterial Activity of Silver Nanoparticles against *Staphylococcus warneri* Synthesized Using Endophytic Bacteria by Photo-irradiation

**DOI:** 10.3389/fmicb.2017.01090

**Published:** 2017-06-14

**Authors:** Zhou-Yan Dong, Manik Prabhu Narsing Rao, Min Xiao, Hong-Fei Wang, Wael N. Hozzein, Wei Chen, Wen-Jun Li

**Affiliations:** ^1^State Key Laboratory of Biocontrol and Guangdong Provincial Key Laboratory of Plant Resources, School of Life Sciences, Sun Yat-Sen UniversityGuangzhou, China; ^2^College of Life Science, Liaoning Normal UniversityDalian, China; ^3^China Tobacco Yunnan Industrial Co., Ltd.Kunming, China; ^4^Bioproducts Research Chair, Zoology Department, College of Science, King Saud UniversityRiyadh, Saudi Arabia; ^5^Botany and Microbiology Department, Faculty of Science, Beni-Suef UniversityBeni-Suef, Egypt; ^6^Key Laboratory of Biogeography and Bioresource in Arid Land, Xinjiang Institute of Ecology and Geography, Chinese Academy of SciencesÜrűmqi, China

**Keywords:** silver nanoparticles, endophyte, *Staphylococcus warneri*, antibacterial activity, DNA cleavage study

## Abstract

Diseases caused by *Staphylococcus warneri* have a significant impact on human health. We evaluated the antibacterial activity of silver nanoparticles (synthesized using the endophytic strain SYSU 333150) against *S. warneri*. The strain SYSU 333150 was isolated from the roots of *Borszczowia aralocaspica* Bunge. The 16S rRNA sequence results suggest that SYSU 333150 belongs to the genus *Isoptericola* and is likely a new species. Photo-irradiation was used to synthesize silver nanoparticles, which were characterized using UV-visible spectroscopy, transmission electron microscopy and X-ray diffraction. The nanoparticles were spherical and measured to be11 to 40 nm. X-ray diffraction revealed four peaks corresponding to the 111, 200, 220, and 311 planes of the face-centered cubic lattice, indicating a crystalline nature. Fourier transform infrared spectroscopy suggested that the metabolites in the culture supernatant were likely reducing and capping agents. The silver nanoparticles possessed antimicrobial activity (14 mm zone of inhibition) against *S. warneri*, which was likely a result of DNA cleavage. The synthesized silver nanoparticles have potent antibacterial activity against *S. warneri* and can be used to control infection.

## Introduction

*Staphylococcus warneri* is a Gram-positive clinically important pathogen that is found on human and animal skin. Although *S. warneri* represents less than 1% of the total staphylococcal population, it is responsible for a wide range of human diseases, such as immune-suppression, infections of the skin, eyes, and urinary tract, and nosocomial infections in immune-compromised patients and neonates (Incani et al., 2010; Legius et al., 2012). To prevent *S. warneri* infections, we evaluated the antibacterial activity of silver nanoparticles.

Nanoparticles are small materials that are less than 100 nanometers in size. They have garnered attention over the past few years because of their potential impact in many areas of science (Manikprabhu and Li, 2015). Metallic nanoparticles are utilized in many fields of science and preliminary results have been encouraging. In particular, silver nanoparticles have received interest because of their unique properties, including chemical stability, good conductivity and catalytic capabilities that can be incorporated into composite fibers, cryogenic superconducting materials, cosmetic products, and electronic components (Ahmed et al., 2016). They also possess antimicrobial properties, which are extensively used to prepare medicine, devices, implants, and dressing materials (Manikprabhu and Lingappa, 2014; Rajesh Kumar et al., 2016). Various physical and chemical methods have been used to synthesize silver nanoparticles, although they involve hazardous chemicals have low material conversions and high energy requirements (Veerasamy et al., 2011).

Nanoparticle synthesis using plants and microbes is currently under investigation (Shahverdi et al., 2007; Ahmed et al., 2016). It is well known that plants synthesize nanoparticles; however, the use of endangered plant species may pose risks and cause an imbalance in plant diversity. Microorganisms are advantageous because they are reliable and inexhaustible (Baker et al., 2015). Microorganisms have been investigated for their nanoparticle synthesis capabilities since the work of Klaus et al. (1999), who first synthesized silver nanoparticles from *Pseudomonas stutzeri* AG259. The soil has been extensively explored as an ecological niche that harbors microorganisms that are potential bio-factories for nanoparticle synthesis (Das et al., 2014; Velusamy et al., 2016).

Apart from soil, microbe-harboring plants comprise one of the richest microbial niches that secrete bioactive compounds (Baker et al., 2015). Recently, research has focused on the isolation of plant endophytes for the synthesis of silver nanoparticles (Qian et al., 2013; Netala et al., 2016). However, only a few reports describe the synthesis of silver nanoparticles using endophytes. Silver nanoparticles from endophytes were used as antimicrobial agents against various pathogens such as *Escherichia coli, P. aeruginosa, S. aureus, Salmonella typhi*, and *Klebsiella pneumoniae* (Sunkar and Nachiyar, 2012). The present study explored the synthesis of silver nanoparticles using bacteria isolated from the *Borszczowia aralocaspica* Bunge plant and evaluated its antibacterial activity against *S. warneri.*

## Materials and Methods

### Isolation and Identification of Endophytic Strain SYSU 333150

Endophytic strain SYSU 333150 was isolated from the roots of *B. aralocaspica* Bunge using International *Streptomyces* project (ISP) 2 medium (Shirling and Gottlieb, 1966). To isolate bacteria, roots were surface sterilized following Khieu et al. (2015). Genomic DNA extraction and PCR amplification of the 16S rRNA gene of SYSU 333150 was performed using a standard protocol (Li et al., 2007). The sequences were compared with available 16S rRNA gene sequences of cultured species from the EzTaxon-e server^1^ (Yoon et al., 2017).

A phylogenetic tree was constructed using the neighbor-joining (NJ) method (Saitou and Nei, 1987) using Kimura’s two parameter model (Kimura, 1980) by MEGA 5.0 software (Tamura et al., 2011) after aligning sequences with CLUSTAL_X (Thompson et al., 1997). Bootstrap analysis was performed with 1,000 replications (Felsenstein, 1985).

### Synthesis of Silver Nanoparticles

Strain SYSU 333150 was grown in 100 ml of ISP 2 broth (Shirling and Gottlieb, 1966) that was incubated for 24 h at 180 rpm and 37°C. After incubation, the broth was centrifuged at 10,000 rpm for 10 min. The supernatant was used for the synthesis of silver nanoparticles. To synthesize silver nanoparticles, the volume of the AgNO_3_ and supernatant was optimized by mixing different volumes of AgNO_3_ (0.002 M) with different volumes of culture supernatant. The optimized protocol included 20 ml of AgNO_3_ combined with 0.5 ml culture supernatant which was then exposed to sunlight for different periods. Silver nanoparticles were also synthesized in the absence of light.

### Characterization of Silver Nanoparticles

Preliminary synthesis of silver nanoparticles was confirmed when the color of the solution changed from colorless to brown (Manikprabhu and Lingappa, 2013). The synthesis was further validated using UV-visible spectroscopy (T60UV-VIS Spectrophotometers). The size and shape of nanoparticles were determined using transmission electron microscopy (TEM). The samples for TEM were prepared by centrifuging the synthesized nanoparticles at 10,000 rpm for 10 min. The supernatant was discarded, washed and resuspended in sterilized distilled water. The prepared solutions were loaded on copper grids for 1 min and the extra solution was removed using blotting paper. The copper grid was then visualized under TEM (JEM-100CX-II).

The crystalline nature of silver nanoparticles was determined using a Rigaku Ultima 4X-ray diffractometer. The radiation used was Cu*Kα* (0.154 nm wavelength) with a scanning rate of 5°/min. To identify the reducing agent in the synthesis of silver nanoparticles, Fourier transform infrared spectroscopy (FTIR) was performed. The spectrum was recorded in the range of 4000–600 cm^-1^ using EQUINOX 55 Perkin Elmer spectrophotometer operated at a resolution of 4 cm^-1^. The FTIR peaks were identified and expressed in wave numbers (cm^-1^).

### Antibiotic Susceptibility, Antibacterial, and DNA Cleavage Study

The antibiotic susceptibility of *S. warneri* (ATCC 27836) was evaluated by the disk diffusion method using Mueller Hinton agar. The antibacterial activity of silver nanoparticles against *S. warneri* (ATCC 27836) was evaluated using the agar well diffusion method (Perez et al., 1990). A 6 mm diameter well was punched on Mueller Hinton agar (containing a pre-lawn of *S. warneri* ATCC 27836) using a sterilized cork borer and was filled with 100 μl of silver nanoparticles (0.53 g/100 ml). The plate was incubated at 37°C for 24 h and the zone of inhibition was recorded. Sterilized distilled water and culture supernatant were used as a control.

To understand the mechanism of antibacterial activity, we performed a DNA cleavage experiment. DNA from *S. warneri* (ATCC 27836) was extracted with our standard protocol (Li et al., 2007). The extracted DNA (5 μl) was mixed with different volumes of silver nanoparticles (5, 10, and 15 μl) and incubated at 37°C for 60 min (at 10 min intervals). Five microliters of *S. warneri* (ATCC 27836) DNA was used as a control. After incubation, the samples were run on a 1.0% agarose gel (in 1 × TAE buffer at 100 V).

## Results

### Identification of Endophytic Strain SYSU 333150

**FIGURE 1:**
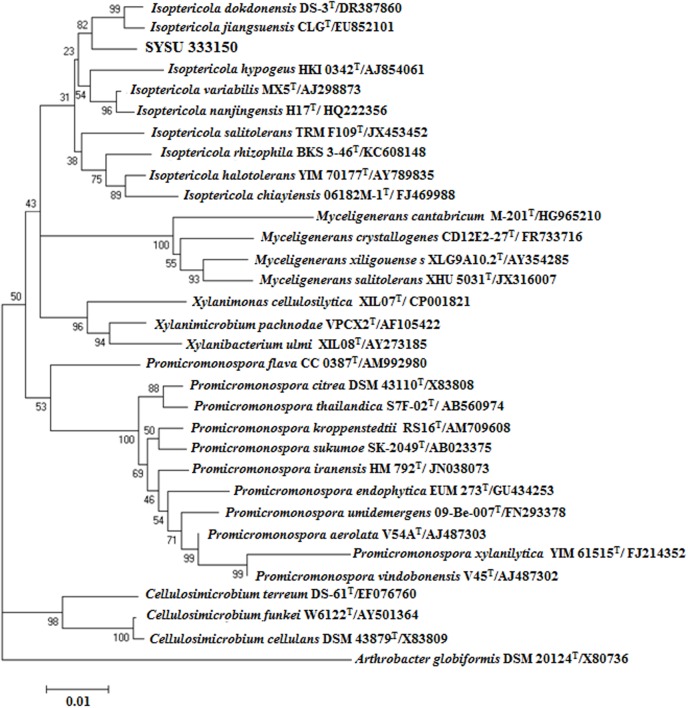
Neighbor-joining phylogenetic tree based on 16S rRNA gene sequences of strain SYSU 333150 and its closest relatives. Bootstrap values are expressed as percentages of 1,000 replications. Bar, 0.01, represents substitutions per site.

Endophytic strain SYSU 333150 was isolated from the roots of *B. aralocaspica* Bunge. An almost complete 16S rRNA sequence (1500 bp) was obtained and subjected to BLAST analysis. Our results showed that strain SYSU 333150 shared 98.6% similarity with *Isoptericola dokdonensis*. The NJ phylogenetic tree based on 16S rRNA sequences indicated that SYSU 333150 formed a clade with the members of the *Isoptericola* genus (**Figure 1**).

### Synthesis and Characterization of Silver Nanoparticles

**FIGURE 2:**
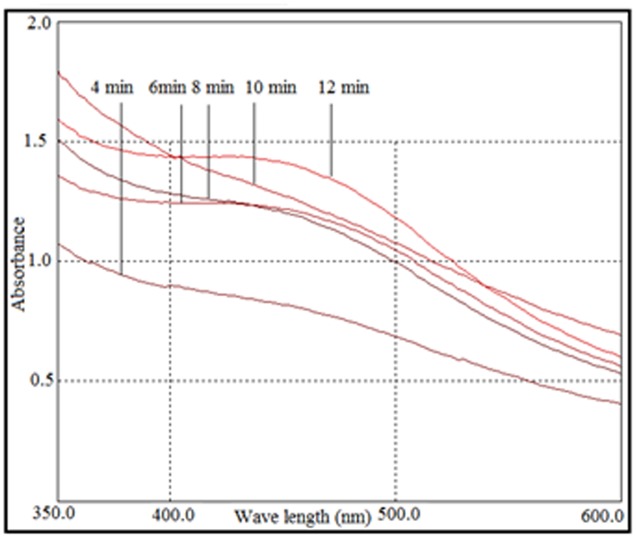
UV-visible spectra of photo irradiation-based synthesis of silver nanoparticles at different time periods.

**FIGURE 3:**
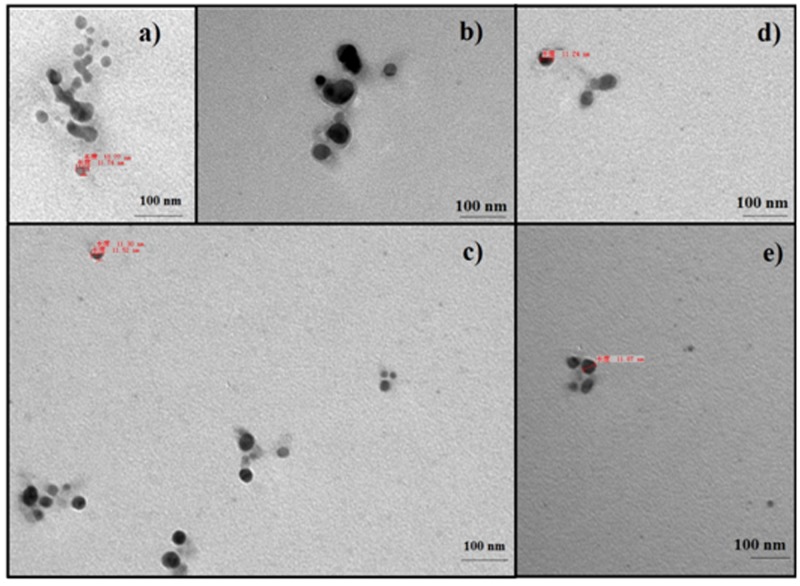
Transmission electron microscopy images of silver nanoparticles: **(a)** 4 min, **(b)** 6 min, **(c)** 8 min, **(d)** 10 min, and **(e)** 12 min.

**FIGURE 4:**
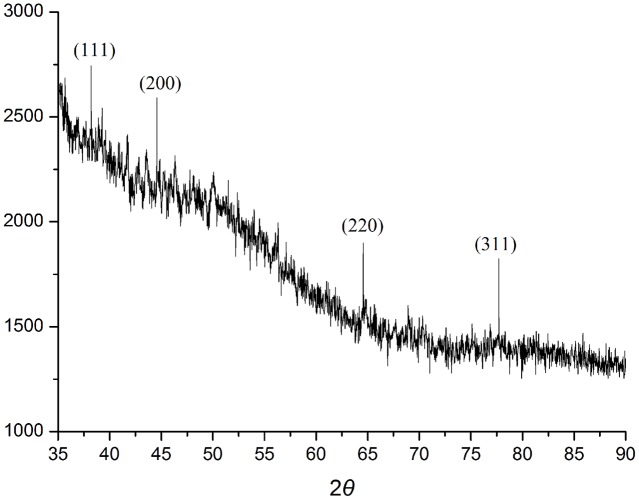
XRD spectra of silver nanoparticles.

**FIGURE 5:**
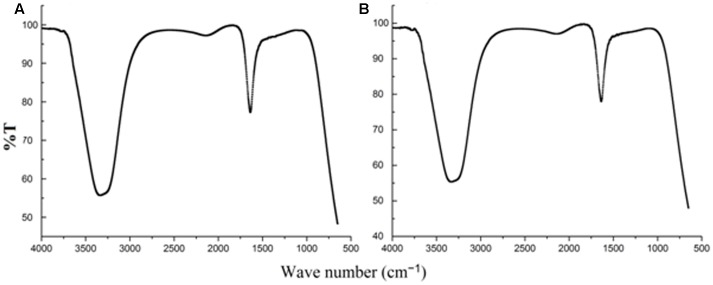
Fourier transform infrared spectroscopy spectra of **(A)** culture supernatant **(B)** silver nanoparticles.

When AgNO_3_ (20 ml) was combined with the supernatant (0.5 ml) and exposed to sunlight, the solution changed from colorless to brown after 4 min. The UV-visible spectra (**Figure 2**) revealed a peak between 400 and 450 nm which increased steadily with reaction time. In the absence of sunlight, silver nanoparticles synthesis was also observed, but only after 3 h (**Supplementary Figure S1**). **Figure 3** shows the TEM images of synthesized silver nanoparticles, which suggest that they were spherical and 11 to 40 nm in size. The X-ray diffraction pattern of the silver nanoparticles show four diffraction peaks (**Figure 4**) at 38.31, 44.58, 64.71, and 77.71°, which correspond to 111, 200, 220, and 311 planes, respectively. **Figure 5** shows the FTIR spectra of the culture supernatant of SYSU 333150 with silver nanoparticles. Both spectra were similar and had absorption peaks at 3200–3400 and 1600–1700 cm^-1^that resulted from OH and C = O groups, respectively.

### Antibiotic Susceptibility, Antibacterial Activity, and DNA Cleavage Experiments

*Staphylococcus warneri* (ATCC 27836) susceptibility to antibiotics was evaluated by the disk diffusion method. **Table 1** shows the antibiotic susceptibility results of *S. warneri* (ATCC 27836). The largest zone of inhibition was observed with norfloxacin and the smallest with oxacillin.

**Table 1:** Antibiotic susceptibility of *S. warneri* (ATCC 27836).

SL. NO.	Antibiotic	Concentration	Zone of inhibition
1.	Penicillin G	10 IU	5 mm
2.	Oxacillin	1 μg	4 mm
3.	Vancomycin	30 μg	8 mm
4.	Amikacin	30 μg	10 mm
5.	Kanamycin	30 μg	7 mm
6.	Tetracycline	30 μg	15 mm
7.	Ciprofloxacin	5 μg	17.5 mm
8.	Chloramphenicol	30 μg	13 mm
9.	Norfloxacin	10 μg	18 mm

**FIGURE 6:**
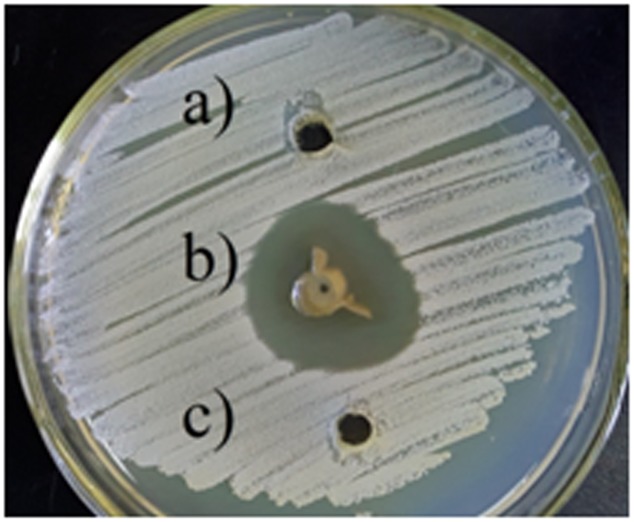
Antibacterial activity of silver nanoparticles against *S. warneri* (ATCC 27836), **(a)** sterilized distilled water, **(b)** silver nanoparticles, and **(c)** culture supernatant.

The antibacterial activity of silver nanoparticles against *S. warneri* (ATCC 27836) resulted in a zone inhibition of 14 mm (**Figure 6**). No zone of inhibition was observed in the control.

**FIGURE 7:**
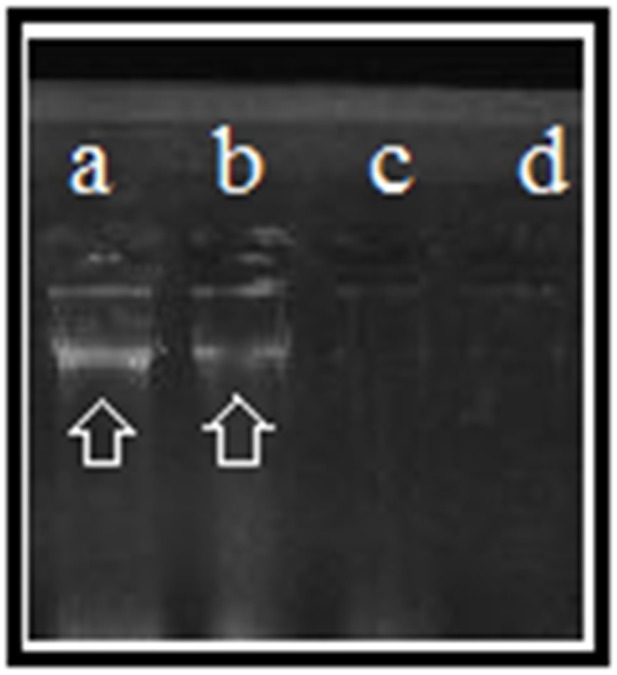
DNA cleavage: **(a)**, control [5 μl of *S. warneri* DNA (ATCC 27836)]; **(b)** [5 μl of *S. warneri* DNA (ATCC 27836) + 5 μl of silver nanoparticles]; **(c)** [5 μl of *S. warneri* DNA (ATCC 27836) + 10 μl of silver nanoparticles]; and **(d)** [5 μl of *S. warneri* DNA (ATCC 27836) + 15 μl of silver nanoparticles]. Arrows indicate the presence of DNA.

**FIGURE 8:**
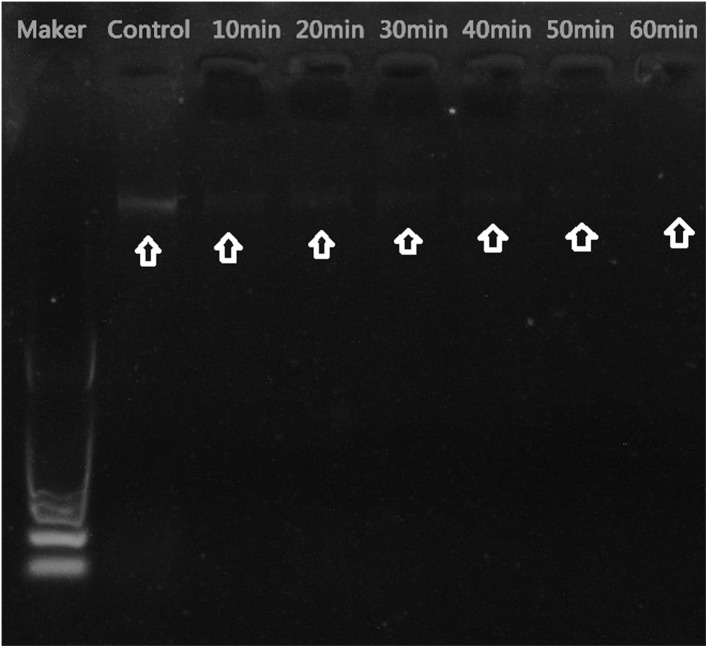
Effect of time on DNA cleavage. Arrows indicate the presence or absence of DNA.

The mechanism of antibacterial activity of silver nanoparticles against *S. warneri* (ATCC 27836) was evaluated using a DNA cleavage experiment. **Figure 7** shows the gel image demonstrating DNA cleavage. The control contained a single band (lane a), whereas partial cleavage was observed in lane b, which contained 5 μl of silver nanoparticles. In lane c and d (10 and 15 μl of silver nanoparticles, respectively) there was complete cleavage of DNA. **Figure 8** shows the effect of time on DNA cleavage; the DNA completely disappeared after 60 min.

## Discussion

Endophytes have demonstrated their capacity to control pathogens, insects and nematodes. They play a crucial role in accelerating the seedling rate and plant growth as well as promote plant establishment under adverse conditions (Sunkar and Nachiyar, 2012). Many studies have investigated endophyte synthesis of silver nanoparticles (Qian et al., 2013; Netala et al., 2016). There are no previous reports of silver nanoparticle synthesis using endophytes from *B. aralocaspica* Bunge. This study assessed the ability of *B. aralocaspica* Bunge endophytes to produce silver nanoparticles.

The endophytic strain SYSU 333150 was isolated from the roots of *B. aralocaspica* Bunge. The 16S rRNA sequence data (98.6% similarity to *I. dokdonensis*) showed that SYSU 333150 belonged to the genus *Isoptericola.* Strain SYSU 333150 also formed a clade with the members of the genus *Isoptericola* in the NJ tree (**Figure 1**). However, the low sequence similarity (>99%) suggested that SYSU 333150 may be a new species in genus *Isoptericola* (Wayne et al., 1987). We hope to further characterize SYSU 333150 to classify its taxonomic position.

The synthesis of silver nanoparticles was indicated when the solution (20 ml AgNO_3_ and 0.5 ml supernatant) changed from colorless to brown (Manikprabhu and Lingappa, 2013) after 4 min of sunlight exposure. In the absence of sunlight, synthesis occurred but took much longer [after 3 h (**Supplementary Figure S1**)]. The sunlight acted as a catalyst, due to photo-excitation, which creates hot electron-hole pairs that are produced by excited molecules. The hot electron-hole pairs were then transferred to surface-adsorbed reducing agent excess electrons, free radicals (such as $O_{2}^{∙-}$) and reduced silverions, which resulted in neutralized silver atoms (Ulug et al., 2015; Manikprabhu et al., 2016).

Similar to our study, Sunkar and Nachiyar (2012) reported silver nanoparticles synthesis (incubation time 72–120 h) from the endophytic bacteria *Bacillus cereus* isolated from *Garcinia xanthochymus*. In the present study, in the absence of sunlight, strain SYSU 333150 produced silver nanoparticles after 3 h. However, sunlight exposure reduced the time to 4 min. These conditions could be useful for the rapid synthesis of silver nanoparticles. Furthermore, SYSU 333150 produced 0.53 g/100 ml silver nanoparticles at a much higher rate (0.14–0.156 g/100 ml) than photo-irradiation synthesis (Manikprabhu and Lingappa, 2013, 2014; Manikprabhu et al., 2016).

The synthesis of silver nanoparticles was further confirmed by UV-visible spectroscopy. The UV-visible spectra (**Figure 2**) revealed a peak between 400 and 450 nm that was caused by surface plasmon resonance of silver nanoparticles in the visible region (Manikprabhu and Lingappa, 2013). The intensity of the peak increased with sunlight exposure, which was possibly due to an increase in silver nanoparticles. After 12 min, the absorption did not increase, suggesting a complete conversion of AgNO_3_ to silver nanoparticles.

The endophytic strain SYSU 333150 produced spherical silver nanoparticles that were11 to 40 nm (**Figure 3**). The particles in this range are well known for having excellent antimicrobial activity (Manikprabhu and Lingappa, 2013, 2014).

The X-ray diffraction pattern of the silver nanoparticles shows four diffraction peaks corresponding to 111, 200, 220, and 311 planes (**Figure 4**). The peaks were consistent with earlier reports of silver nanoparticles that were synthesized by endophytes. These data suggest thatthe silver nanoparticles are crystalline in nature and have a face-centered cubic (FCC) phase (Sunkar and Nachiyar, 2012; Qian et al., 2013; Netala et al., 2016).

The FTIR spectra (**Figure 5**) of SYSU 333150 supernatant and silver nanoparticles were similar and had absorption peaks at 3200–3400 and 1600–1700 cm^-1^that were caused by OH and C = O groups. It is possible that these peaks result from the reducing and capping agents that are responsible for silver nanoparticle synthesis. It has been reported that biological molecules can perform dual functions (formation and stabilization of silver nanoparticles) in aqueous medium (Sunkar and Nachiyar, 2012).

*Staphylococcus warneri* causes many infections (Incani et al., 2010; Legius et al., 2012). We evaluated the antimicrobial activity of silver nanoparticles against *S. warneri* (ATCC 27836). Silver nanoparticles synthesized by the endophytic strain SYSU 333150 demonstrated antimicrobial activity (zone of inhibition of 14 mm). Antibacterial activity of silver nanoparticles against *S. warneri* was also reported by El-Batal et al. (2013) although the nanoparticles were synthesized using *B. stearothermophilus* and gamma radiation.

There are several proposed mechanisms of antibacterial activity for silver nanoparticles. These mechanisms include depletion of intracellular ATP through destabilization of the outer membrane, rupture of the plasma membrane, blocking of respiration by reacting with sulfhydryl (–S–H) groups along the cell wall to form R–S–S–R bonds and DNA damage (Manikprabhu and Lingappa, 2013; Rajesh Kumar et al., 2016). To determine the mechanism of antibacterial activity, we performed a DNA cleavage experiment. Our results suggest that the concentration of silver nanoparticles as well as the incubation time affects DNA cleavage. Fifteen microliters of silver nanoparticles resulted in complete cleavage of DNA (**Figure 7**). Incubation time also affected DNA cleavage. Initially, there was no DNA cleavage (**Figure 8**). However, over time, the DNA started to fade and completely disappeared after 60 min. DNA cleavage may be a result of the interaction of silver nanoparticles with sulfur and phosphorous present in DNA (Sathish Kumar et al., 2009).

## Conclusion

We conclude that photo-irradiation with the endophytic strain SYSU 333150 is a rapid and efficient method to synthesize spherical silver nanoparticles. The synthesized silver nanoparticles exhibited antimicrobial activity against *S. warneri* through DNA cleavage. The synthesized nanoparticles can used in biomedical applications to treat diseases caused by *S. warneri.*

## Author Contributions

Z-YD and MPNR planned, conducted the experiments, analyzed the data, and prepared the manuscript. MX and WH revised the manuscript. H-FW isolated the strain SYSU 33150. WC and W-JL supervised the experiment.

## Conflict of Interest Statement

The authors declare that the research was conducted in the absence of any commercial or financial relationships that could be construed as a potential conflict of interest.

## Footnotes

1 http://www.ezbiocloud.net/

## Supplementary Material

FIGURE S1 UV-visible spectra of silver nanoparticles synthesized in the absence of sunlight.
