# Supplementary figures and images for: Antibacterial Activity of Silver Nanoparticles against Staphylococcus warneri Synthesized Using Endophytic Bacteria by Photo-irradiation
Source: Front Microbiol. 2017 Jun 14;8:1090. doi: 10.3389/fmicb.2017.01090 (PMC5469913; doi:10.3389/fmicb.2017.01090)

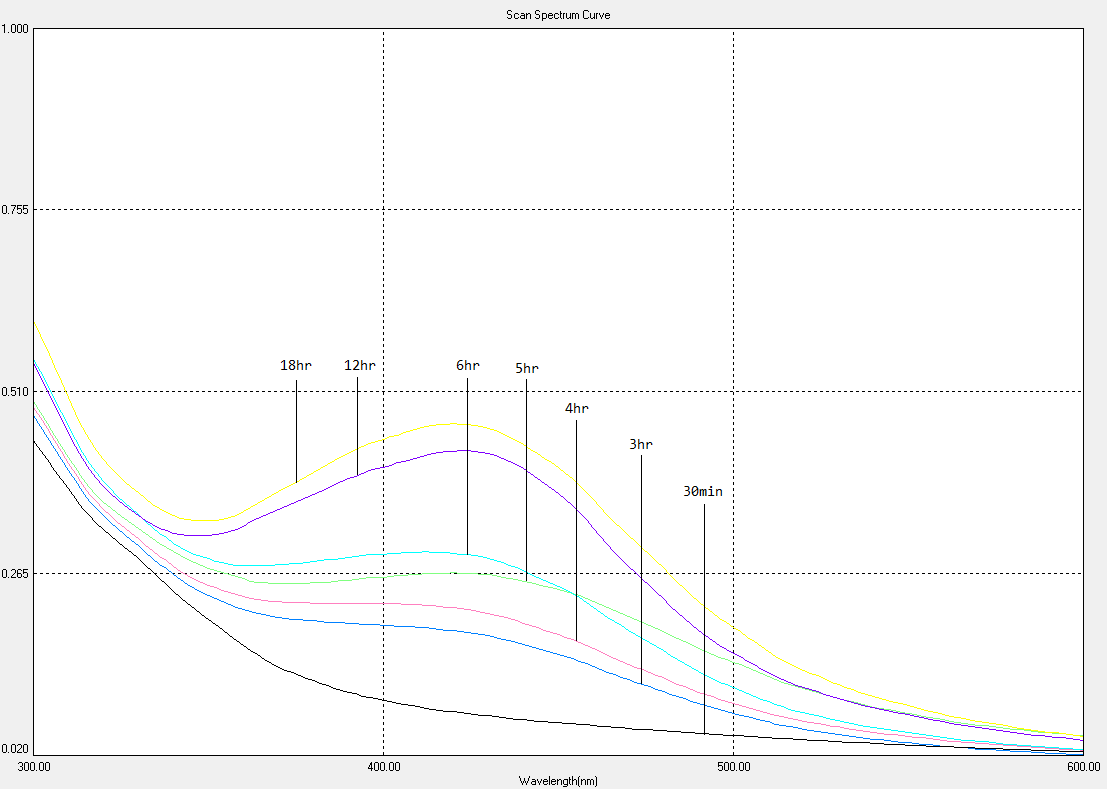

Supplement: FIGURE S1 — UV-visible spectra of silver nanoparticles synthesized in the absence of sunlight. [file Image_1.TIF]
